# Cell‐free DNA aneuploidy score as a dynamic early response marker in prostate cancer

**DOI:** 10.1002/1878-0261.13797

**Published:** 2025-03-14

**Authors:** Khrystany T. Isebia, Anouk C. de Jong, Lisanne F. van Dessel, Vanja de Weerd, Corine Beaufort, Jean Helmijr, José Alberto Nakauma‐González, Job van Riet, Paul Hamberg, Daniel Vis, Michiel S. van der Heijden, Nick Beije, Martijn P. Lolkema, Teoman Deger, Saskia M. Wilting, Ronald de Wit, Maurice P. H. M. Jansen, John W. M. Martens

**Affiliations:** ^1^ Department of Medical Oncology and Cancer Genomics Netherlands, Erasmus MC Cancer Institute University Medical Center Rotterdam The Netherlands; ^2^ Department of Urology, Erasmus MC Cancer Institute University Medical Center Rotterdam The Netherlands; ^3^ Division of AI in Oncology German Cancer Research Center (DFKZ) Heidelberg Germany; ^4^ Department of Internal Medicine Franciscus Gasthuis & Vlietland Rotterdam The Netherlands; ^5^ Department of Medical Oncology The Netherlands Cancer Institute‐Antoni van Leeuwenhoek Hospital Amsterdam The Netherlands

**Keywords:** abiraterone, cabazitaxel, ctDNA, docetaxel, enzalutamide, prostate cancer

## Abstract

Cell‐free circulating tumor DNA (ctDNA) has emerged as a promising biomarker for response evaluation in metastatic castration‐resistant prostate cancer (mCRPC). The current study evaluated the modified fast aneuploidy screening test‐sequencing system (mFast‐SeqS), a quick, tumor‐agnostic and affordable ctDNA assay that requires a small input of DNA, to generate a genome‐wide aneuploidy (GWA) score in mCRPC patients, and correlated this to matched metastatic tumor biopsies. In this prospective multicenter study, GWA scores were evaluated from blood samples of 196 mCRPC patients prior to treatment (baseline) with taxanes (docetaxel and cabazitaxel) and androgen receptor signaling inhibitors (ARSI; abiraterone and enzalutamide), and from 74 mCRPC patients at an early timepoint during treatment (early timepoint; median 21 days). *Z*‐scores per chromosome arm were tested for their association with tumor tissue genomic alterations. We found that a high tumor load in blood (GWA^high^) at baseline was associated with poor response to ARSI [HR: 2.63 (95% CI: 1.86–3.72) *P* < 0.001] but not to taxanes. Interestingly, GWA^high^ score at the early timepoint was associated with poor response to both ARSIs [HR: 6.73 (95% CI: 2.60–17.42) *P* < 0.001] and taxanes [2.79 (95% CI: 1.34–5.78) *P* = 0.006]. A significant interaction in Cox proportional hazards analyses was seen when combining GWA status and type of treatment (at baseline *P* = 0.008; early timepoint *P* = 0.018). In summary, detection of ctDNA in blood by mFast‐SeqS is cheap, fast and feasible, and could be used at different timepoints as a potential predictor for outcome to ARSI and taxane treatment in mCRPC.

## Graphical abstract

mFast‐SeqS‐based genome‐wide aneuploidy scores are concordant with aneuploidy scores obtained by whole genome sequencing from tumor tissue and can predict response to ARSI treatment at baseline and, at an early time point, to ARSI and taxanes. This assay can be easily performed at low cost and requires little input of cfDNA.

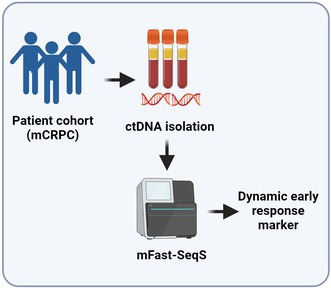

**Abbreviations**
ARSI: androgen receptor signaling inhibitor
CN: copy number
CS: cellsave
ctDNA: cell‐free circulating tumor DNA
dPCR: digital PCR
FFS: failure‐free survival
GWA: genome‐wide aneuploidy
HMF: Hartwig Medical Foundation
IQR: interquartile range
mCRPC: metastatic castration‐resistant prostate cancer
mFast‐SeqS: modified fast aneuploidy screening test‐sequencing system
OS: overall survival
REMARK: recommendations for tumor marker prognostic studies
SD: standard deviation
TTNT: time to next treatment line
WGS: whole genome sequencing

## 1 Introduction

With the increasing number of available therapies for patients with metastatic castration‐resistant prostate cancer (mCRPC), accurate response prediction has become increasingly important [1, 2, 3, 4, 5]. Currently available blood‐based biomarkers, such as prostate specific antigen (PSA), have limitation in assessing response in patients with mCRPC [6, 7]. Cell‐free circulating tumor DNA (ctDNA) has emerged as a promising biomarker with diagnostic, predictive and prognostic applications in cancer [8, 9, 10, 11, 12, 13]. The fraction of ctDNA within the total pool of cell‐free DNA (cfDNA) has been associated with tumor burden and may be used to predict and monitor response to treatment [12]; however, prior prostate cancer studies were hampered by inconsistent sampling and studies to test whether baseline and on‐treatment ctDNA fraction can predict response to treatment are lacking. Most techniques to measure the fraction of ctDNA rely on prior knowledge of genomic aberrations determined in a tumor biopsy. Therefore, they are dependent of upfront deep sequencing of tumor tissue, which is time consuming and costly.

The modified Fast aneuploidy screening test‐sequencing system (mFast‐SeqS) was originally established as a noninvasive screening method for fetal aneuploidy in maternal blood [14, 15]. mFast‐SeqS uses unique LINE‐1 elements scattered across the human genome, the aneuploidy of a cfDNA sample can be estimated globally using *z*‐statistics without prior knowledge of the genomic make‐up of the tumor *in situ* [16]. If this score, termed the genome‐wide aneuploidy (GWA) score, is larger than 5, the tumor fraction in cfDNA is usually larger than 10% [14]. This way, the mFast‐SeqS‐based GWA score serves as a fast, easily applicable, and affordable method to estimate the tumor fraction in cfDNA and only requires a minute amount of cfDNA (0.5−1 ng). Therefore, mFast‐SeqS may be a promising technique for response evaluation in patients with mCRPC in daily clinical practice. This technique has been used by our group before in mCRPC in which we validated the GWA score as a prognostic biomarker in prostate cancer [17]. The current study aimed to evaluate the clinical value of aneuploidy in mCRPC patients using mFast‐SeqS. Longitudinal cfDNA samples and metastatic tumor biopsies were collected in mCRPC patients treated with androgen receptor signaling inhibitors (ARSI) or taxanes, and these samples were assessed for the GWA score at baseline and during treatment. Additionally, the concordance between the copy number alterations (CNA) of tumor tissue biopsy and ctDNA was evaluated in matched ctDNA‐tumor samples.

## 2 Materials and methods

### 2.1 Patient inclusion

The current analysis encompasses patients with mCRPC treated with abiraterone, enzalutamide, docetaxel or cabazitaxel and enrolled in the CPCT‐02 study (NCT01855477) with or without simultaneous inclusion in the CIRCUS study (NTR5732) between February 2015 and December 2019. The CPCT‐02 study obtained fresh‐frozen tumor biopsies of a metastatic lesion for whole genome sequencing (WGS). Additionally, baseline and on‐treatment blood biopsies for cfDNA were drawn. In‐ and exclusion criteria have been published before [18]. In short, patients were eligible if they had a locally advanced or metastatic solid tumor for which a next line of systemic treatment with a registered anti‐cancer agent was indicated and a fresh tumor biopsy of a metastatic lesion could be obtained. The list of participating hospitals is available via www.cpct.nl/ziekenhuizen. Patients with mCRPC enrolled in the CPCT‐02 study could also enter the multicenter, prospective CIRCUS study, in which additional longitudinal blood samples for cfDNA during multiple treatment lines were obtained. The CIRCUS study was conducted at the Erasmus MC, the Franciscus Gasthuis & Vlietland and the HagaZiekenhuis. Both studies were conducted following the Declaration of Helsinki and approved by the medical ethical committee of University Medical Center Utrecht (CPCT‐02) and Erasmus University Medical Center (CIRCUS; MEC‐16‐081). All patients provided written informed consent before any study procedure was performed. Clinical data for these patients were collected in an electronic case report form (ALEA Clinical) Abcoude, the Netherlands.

### 2.2 Study endpoints

The main outcomes were failure‐free survival (FFS) and time to next treatment line (TTNT). For patients treated with ARSI, FFS was defined as the time from the start of treatment until the end of treatment with ARSI. For patients treated with taxanes, the outcome was slightly adapted, as a response to taxanes can continue after the end of treatment. Therefore, TTNT was defined as the time from the start of treatment until the next treatment line, best supportive care or death, whichever occurred first. The study endpoints were reported according to the Reporting Recommendations for Tumor Marker Prognostic Studies (REMARK) [19].

### 2.3 Tissue biopsies, blood sampling, and cfDNA extraction

Image‐guided core needle biopsies of metastatic tumor lesions were obtained before the start of a new line of systemic treatment, as described previously by Priestley et al. [20].

Blood was drawn prior to starting treatment and at an early time‐point (± 3 weeks) after the start of the treatment in one to three CellSave Preservative tubes (CS) (Menarini Silicon Biosystems, Castel Maggiore, Bologna, Italy) and processed within 96 h after withdrawal as described by van Dessel et al. [21].

Plasma was isolated from blood draws by two centrifugations steps of 10 min at room temperature, at 1711 ***g*** and 12 000 ***g***, respectively. Plasma was then stored at −80 °C in 2 mL aliquots until further use for cfDNA isolation. cfDNA was extracted using the Maxwell platform (Maxwell® RSC LV ccfDNA Plasma Custom Kit, Promega, Madison, WI, USA) or QIAsymphony platform (QIAsymphony® (QS) SP Circulating DNA Kit, Qiagen, Venlo, Limburg, The Netherlands) according to the manufacturer's protocol. Afterwards, cfDNA was stored at −20 °C. cfDNA concentrations were quantified using the Quant‐iT dsDNA high‐sensitivity assay (Invitrogen, Life Technologies, Carlsbad, CA, USA) and the Qubit fluorometer (Invitrogen) was used for the readout according to the manufacturer's instructions.

### 2.4 Whole genome sequencing data analysis

Whole genome sequencing data from metastatic tumor tissue (CPCT02 cohort) were provided (data request number DR‐170) by the Hartwig Medical Foundation (HMF), Amsterdam, and were described elsewhere [20]. From the received WGS‐data, additional processing was performed using a workflow as previously reported [22]. For tissue, copy number alterations were estimated using purple v2.49, Hartwig Medical Foundation, Amsterdam, the Netherlands. From these data, the relative copy number deviation from the expected normal copy number was estimated per chromosomal arm and log^2^ transformed.

### 2.5 mFast‐SeqS, sequencing, and data analysis

To obtain copy number profiles, the mFast‐SeqS protocol was performed on 1 ng cfDNA as previously described [14, 23]. Briefly, LINE‐1 elements scattered throughout the genome were amplified using a carefully selected primer pair [15]. Bar‐coded adaptors were added, and the resulting libraries were pooled and sequenced on a MiSeq system generating 150 bp single‐end reads to reach at least 90 000 reads per sample. Read counts per chromosome arm were then normalized to the total library size and then a *Z*‐score per chromosome arm was calculated relative to healthy male controls (*n* = 17). Due to insufficient absolute number LINE‐1 elements, the short arms of chromosomes 13, 14, 15, 21, and 22 as well as the Y chromosome were excluded from analysis. These *Z*‐scores and the log2 tissue‐based CNA per chromosomal arm were compared for correlations as described previously by Mendelaar et al. [22].

Finally, the resulting *Z*‐scores per chromosome were squared and summed yielding a genome‐wide aneuploidy (GWA) score per sample. Based on the previously defined cut‐off set by Belic et al. [16], samples were dichotomized in genome‐wide aneuploidy score (GWA) low (GWA < 5) or high (GWA ≥ 5) designated respectively GWA^low^ or GWA^high^.

### 2.6 Evaluation of chromosome Xq and AR‐amplification

As the mFast‐SeqS assay is not able to detect the AR amplification directly, we verified the AR‐amplification in blood by a more specific assay namely the digital PCR (dPCR). This assay quantified the number of AR genomic copies and compared these to the number of reference gene *AGO1* genomic copies (on chromosome 1p34.3), resulting in an AR/AGO1 copy number (CN)‐ratio similar to the AR/RNaseP CN‐ratio reported by others [24]. The dPCR AR amplification assay was tested on prostate cancer cell lines LNCAP and VCAP, representing normal and amplified AR models, respectively. Afterward, the AR/AGO1 CN‐ratio was determined by dPCR on available blood cfDNA from 82 mCRPC patients with mFast‐SeqS GWA status determined as GWA^high^ (*n* = 35) or GWA^low^ (*n* = 47).

### 2.7 Statistical analysis

Baseline characteristics, clinical outcomes, and GWA scores were described as mean ± standard deviation (SD), median and interquartile range (IQR), or number of events. FFS was associated with dichotomized GWA scores at baseline and the early time point. Next, Kaplan Meier curves for FFS and OS were generated based on the dichotomized GWA scores, and log‐rank tests were performed to determine the statistical significance of the differences between survival curves of subgroups. Hazard ratios and their 95% confidence intervals (95% CI) were determined by Cox regression. Depending on the format and distribution of the data, the appropriate statistical tests were used as described in the figure legends. The *P*‐values were two‐sided, and *P* ≤ 0.05 was considered significant. All statistical analyses were performed using either spss (v.28), SPSS inc, Chicago, IL, USA, stata (v.17), StataCorp, College Station, TX, USA or the statistical platform r (v4.3.0.), R Foundation, Vienna, Austria.

## 3 Results

### 3.1 Samples and baseline characteristics

Between February 2015 and December 2019, 235 patients with mCRPC were included in the CPCT‐02 trial (NCT01855477) and treated directly after a fresh‐frozen tumor biopsy was obtained [18]. The CPCT‐02 study also collected blood before treatment (baseline) from 196 mCRPC patients. WGS was successfully performed in 155/235 (66%) patients. For 153 patients matched WGS and baseline cfDNA analysis using mFast‐SeqS were available. The median age at registration of these included patients for the WGS analysis was 68 years. The median time between biopsy and baseline blood draw was 0 days (range = 0–187 days). The majority of the biopsies (51%) were taken from lymph node metastases, followed by 29.4% from bone metastases. The baseline characteristics of all cohorts are described in Table 1.

**Table 1:** Baseline characteristics.

Characteristics	mFast‐SeqS			WGS (*N* = 153)
	Total (*n* = 196)	ARSI (*n* = 154)	Taxanes (*n* = 42)	
Age at registration				
Median (range), year	68 (46–84)	68.5 (46–84)	67 (55–80)	68 (46–84)
Biopsy site, no. (%)				
Lymph node	70 (35.7%)	59 (38.3%)	11 (26.2%)	78 (51%)
Bone	42 (21.4%)	37 (24%)	5 (11.9%)	45 (29.4%)
Liver	14 (7.1%)	12 (7.8%)	2 (4.8%)	16 (10.5%)
Lung	2 (1.0%)	2 (1.3%)		3 (2.0%)
Soft tissue	10 (5.1%)	9 (5.8%)	1 (5.8%)	9 (5.9%)
Other	3 (1.5%)	3 (1.9%)		2 (1.3%)
Missing	55 (28.1%)	32	23 (54.8%)	0
Time between biopsy and baseline blood draw				
Median (range), days				0 (0–187)
Time between start treatment and baseline blood draw				
Median (range), days	4.5 (0–28)	3.5 (0–28)	6 (0–27)	
Prior therapy lines, no. (%)				
Median (range)	1 (0–9)	1 (0–9)	2 (0–4)	2 (0–9)
Type of prior therapy, no. (%)				
Abiraterone	26 (13.3%)	24 (15.4%)	7 (16.7%)	25 (16.4%)
Enzalutamide	60 (30.6%)	46 (29.5%)	26 (61.9%)	50 (32.7%)
Docetaxel	129 (65.8%)	103 (66%)	29 (69%)	99 (64.7%)
Cabazitaxel	51 (26%)	44 (28.2%)	2 (4.8%)	42 (27.5%)
Radium‐223	18 (9.2%)	16 (10.3%)	1 (2.4%)	18 (11.8%)
Other ARSI	3 (1.9%)	3 (1.9%)	2 (4.8%)	3 (2.0%)
Other chemotherapy	4 (2.0%)	4 (2.6%)		3 (2.0%)
Other systemic treatment	11 (5.6%)	11 (7.1%)	1 (2.4%)	10 (6.5%)
PSA at start treatment, μg·L^−1^				
Median (range)	60 (3.1–2375)	61.5 (3.1–2375)	55 (5–1690)	
Missing	84	66	18	

**Fig. 1:**
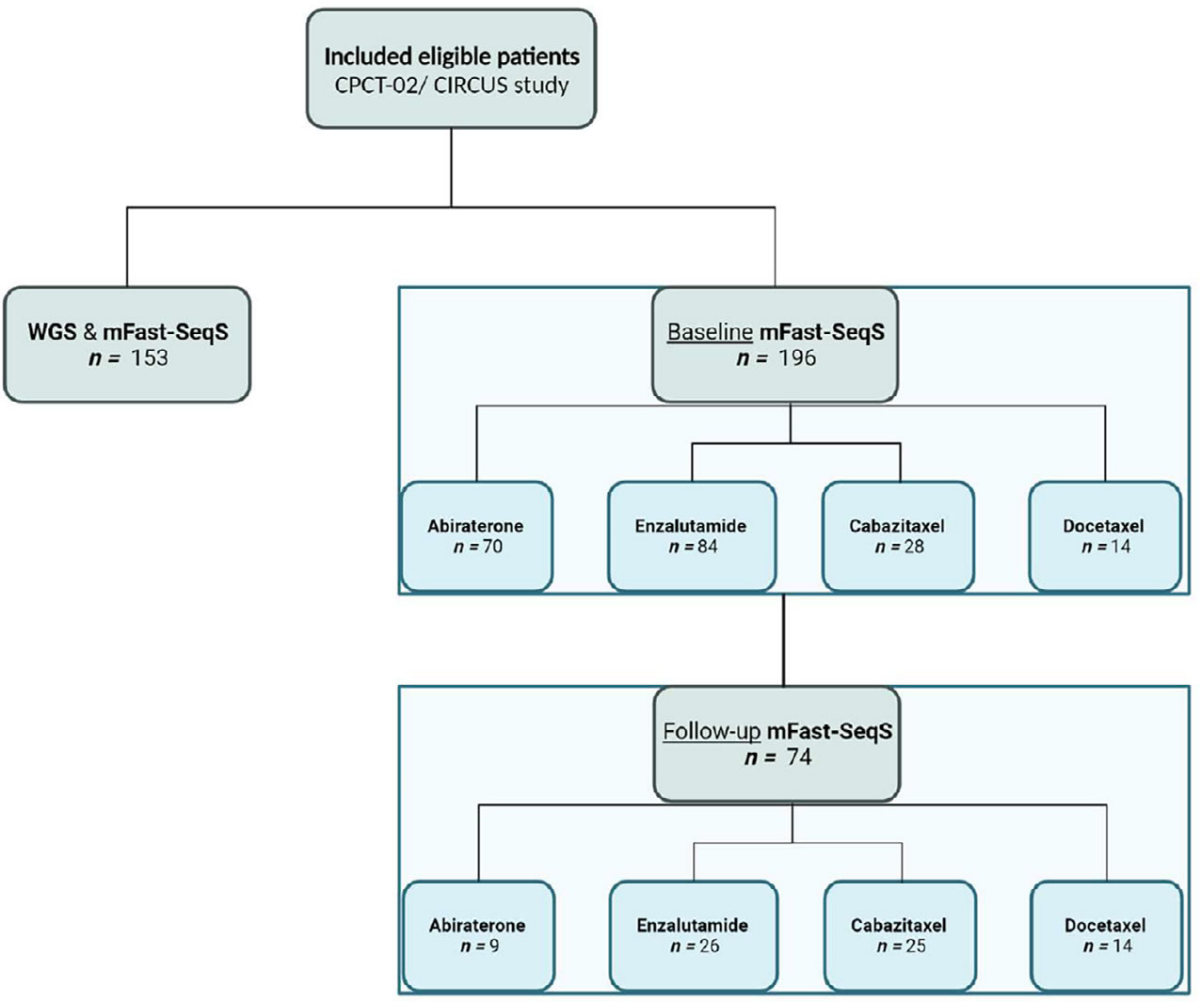
Schematic overview of mCRPC‐patient inclusion within the CPCT‐02 and CIRCUS study. Overview of metastatic castration resistant prostate cancer (mCRPC)‐patient enrollment into the CPCT‐02 and CIRCUS studies and subsequent analyses performed. In total, 196 patients were enrolled prior to a new treatment line with androgen receptor signaling inhibitor (ARSI) or taxanes. Patients could be included multiple times, depending on whether they received multiple new treatment lines. Of the 196 patients, we had 153 unique tissue biopsies available for WGS.

The mFast‐SeqS was performed on blood cfDNA to generate a GWA score per patient, and this was assessed according to the study design shown in the flowchart of Fig. 1. Baseline liquid biopsies were available for analysis with clinical outcomes for 154 patients treated with ARSI and 42 patients treated with taxanes. An early on‐treatment timepoint (median 21 days, IQR: 19–28 days after start of treatment) sample was available for 35 patients treated with ARSI and 39 treated with a taxane. The median age at registration of the included patients was 68 years. Patients had received a median of 1 prior treatment line (excluding ADT) for metastatic prostate cancer, mostly docetaxel (65.8%). The median PSA before the start of treatment was 60 ng·mL^−1^ (range: 3.1–2375).

Median blood GWA scores were 4.7 (IQR: 1.4–35.25) at baseline and 1.22 (IQR:0.3–9.61) at early time point which translated after dichotomization (< 5 vs. ≥ 5) in 95 of 196 patients (48%) and 19 of 74 patients (26%) into being GWA^high^ at baseline and at early time point on treatment, respectively.

### 3.2 Whole genome sequencing of tumor tissue cohort and correlation with GWA

**Fig. 2:**
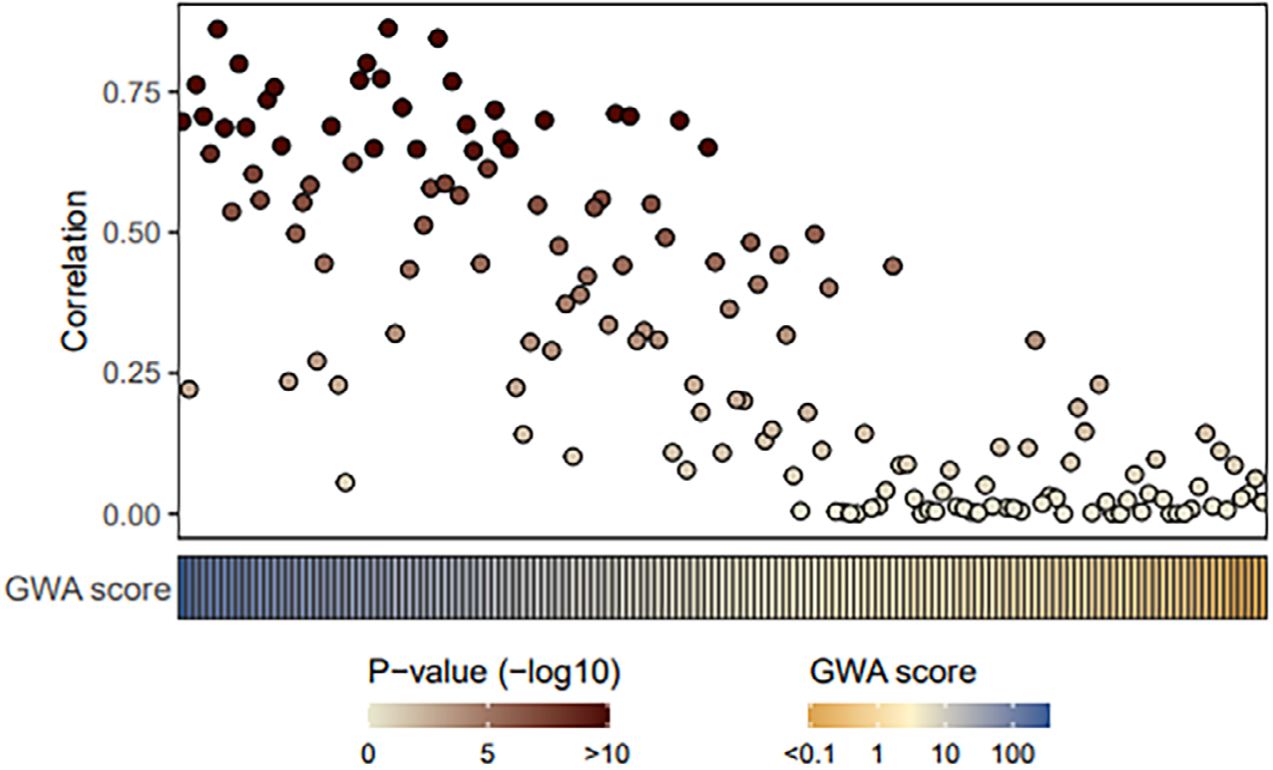
Correlation between GWA score of cfDNA and tumor tissue aneuploidy. Spearman correlation coefficient between tissue‐based copy number alterations (CNA) per chromosome arms derived from WGS (*x*‐axis) and the aneuploidy scores per chromosome arms in cell‐free DNA (cfDNA) derived from mFast‐SeqS data (*y*‐axis). The relative deviation of CNA from the expected normal CNA was calculated and log_2_ transformed and the results are shown per sample. The graphs represent matched samples ordered according to the genome‐wide aneuploidy (GWA) score.

**Fig. 3:**
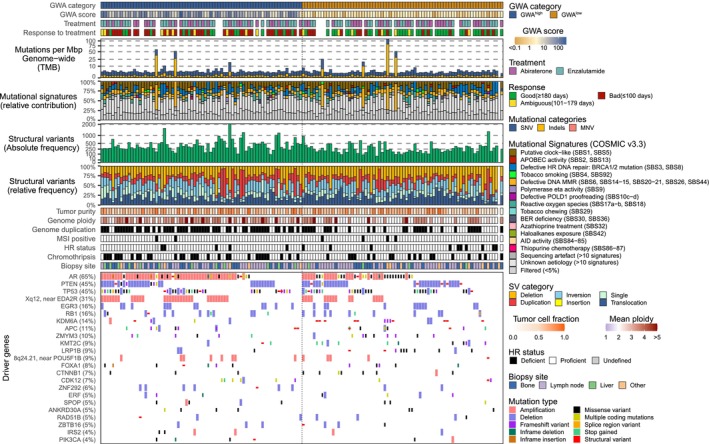
Genomic landscape of included patients (oncoplot), ordered by dichotomized GWA. Overview of genome‐wide tumor tissue characteristics of the cohort (CPCT‐02; *n* = 153) ordered by dichotomized genome‐wide aneuploidy (GWA) derived from cfDNA. From top to bottom, the tracks represent: Dichotomized GWA score in blood; GWA score in blood; type of treatment, if known; response to treatment category; genome‐wide tumor mutational burden (mutations per megabase); the relative contribution of Catalog of Somatic Mutations in Cancer (COSMIC) mutational signatures (v3.3), grouped per proposed etiology; absolute frequency of structural variants; relative frequency of structural variants; tumor purity; genome ploidy; genome duplication detected; microsatellite‐instability (MSI) status; homologous recombination (HR) status as detected by Classifier of HOmologous Recombination Deficiency (CHORD) [25]; presence of chromothripsis; generalized biopsy location; overview of detected somatic mutations in driver genes estimated by dNdScv [26] and GISTIC2 [27].

When comparing the CNA between blood cfDNA and tumor tissue DNA, a statistically significant positive correlation was seen (median Rho of 0.75; range: 0.24–0.93) (Fig. 2, and Figs S1 and S2). We further investigated whether GWA^high^ patients when compared to GWA^low^ had statistically significant different tumor characteristics (Oncoplot Fig. 3). Patients with blood GWA^high^ had a significantly higher genome ploidy (*P* < 0.001), a higher number of structural variants (*P* < 0.001), and more whole genome duplications (*P* < 0.001) in their tumor tissue when compared to tumor tissue of patients with blood GWA^low^. Mutation analysis of tissue‐based WGS revealed that somatic mutations were present in driver genes in the majority of patients in *AR* (65%) followed by *TP53* (45%), *PTEN* (45%), and *EDA2R* (31%). The GWA^high^ patients had significantly more *AR* (*P* = 0.025) and *EDA2R* amplifications (*P* = 0.001) (both on chromosome Xq11‐q12) in their tumor tissue when compared to GWA^low^ patients. As the number of LINE‐1 elements on the X chromosome is low, AR amplification was evaluated in 82 cfDNA samples with the dPCR. To this end, we used dPCR to quantify AR and a reference gene AGO1 and derived the ratio between these two genes (AR/AGO ratio) as a measure of AR amplification and correlated this measure to GWA score and aneuploidy on Xq (expressed as Xq *Z*‐scores) measured by the mFast‐SeqS (Fig. S3). These analyses showed a trend towards higher AR/AGO1 ratio in GWA^high^ cases compared to those that are GWA^low^ (*P* = 0.08) confirming that GWA^high^ is in part defined by aneuploidy on Xq that coincides with AR amplification. Between the GWA score and AR/AGO1 dPCR ratio a modest correlation was observed (Pearson correlation: 0.47, *R* ^2^ = 0.22) (Fig. S4).

### 3.3 Clinical outcome and relation with GWA

**Fig. 4:**
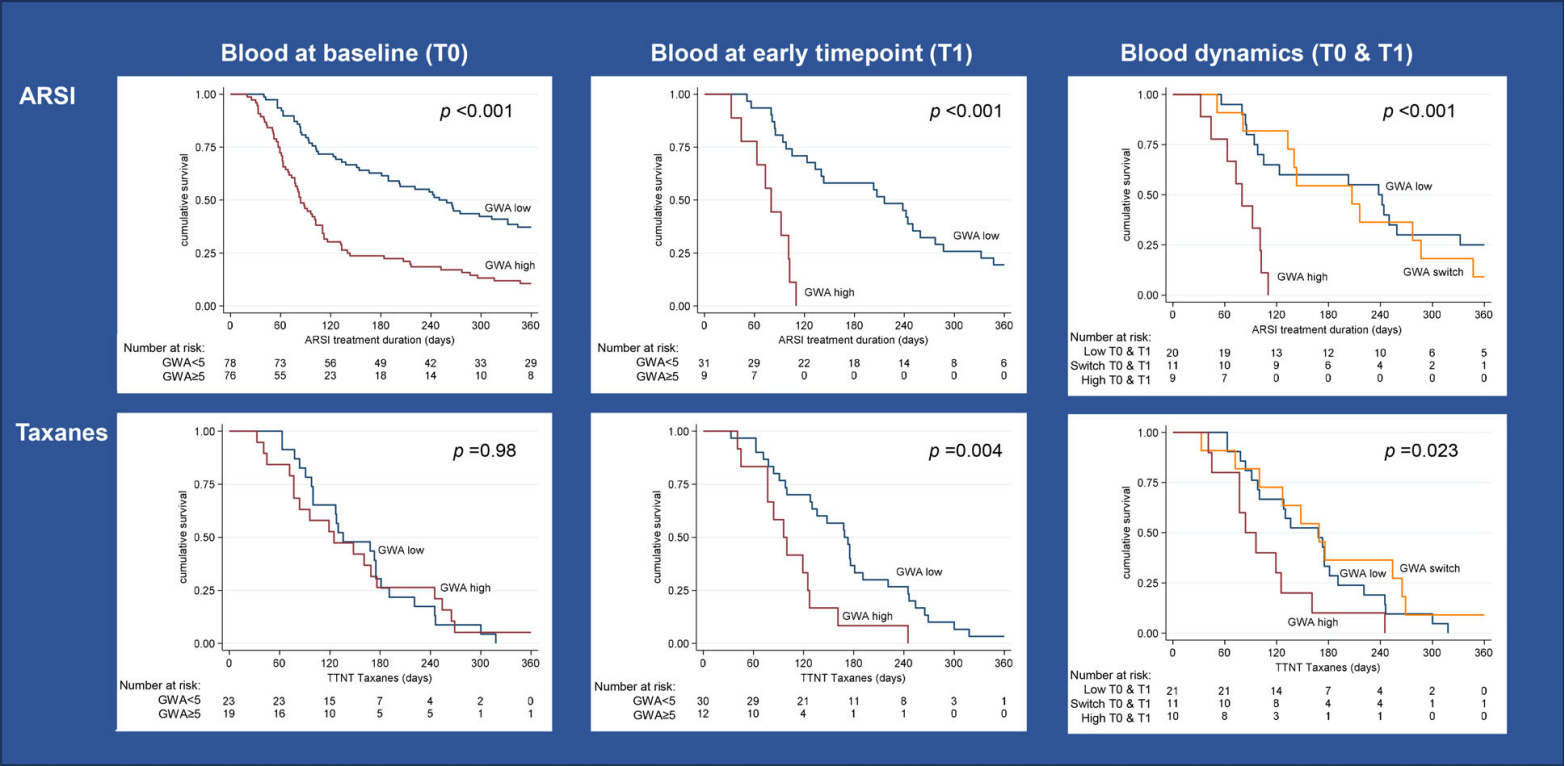
Blood GWA and treatment response. Kaplan Meier curves of failure‐free survival (FFS) were grouped by type of treatment [androgen receptor signaling inhibitor (ARSI) or taxanes] and dichotomized based on blood GWA score. This was done for at baseline timepoint samples, early time point samples and longitudinal timepoint (combined baseline and early on treatment timepoint). Groups based on genome‐wide aneuploidy (GWA) scores were compared using a Log‐Rank test.

We evaluated whether GWA status was associated with clinical outcome. In patients treated with taxanes (cabazitaxel or docetaxel), we found no statistically significant difference in TTNT when comparing between patients with a GWA^low^ and GWA^high^ score for the baseline while at early time point sample in patients with GWA^high^, we observed a shorter TTNT [HR: 2.79 (95% CI: 1.35–5.78) *P* < 0.01] in the cox regression (Table 2) and Kaplan–Meier analyses (Fig. 4). In contrast, for patients that were treated with ARSI already at baseline GWA^high^ predicted a shorter FFS [HR: 2.63 (95% CI: 1.86–3.72) *P* < 0.001], difference that at early time point seemed even more pronounced [HR: 6.73 (95% CI: 2.60–17.42) *P* < 0.001] (Fig. 4 and Table 2). For ARSI treated patients, who were stratified on dichotomized GWA score, Kaplan–Meier analysis revealed a significant difference in FFS (*P* < 0.001), with a median of 280 days and of 120 days for patients with GWA^low^ and GWA^high^, respectively (Fig. 4). Cox proportional hazards analyses for outcome at baseline samples showed a significant interaction between GWA status and treatment type (ARSI vs. taxanes; *P* = 0.008, Table 3) and subsequently, at early timepoint (*P* = 0.018, Table 3).

**Table 2:** Overview of GWA and hazard ratio per treatment.

Treatment	Response	Timepoint blood biopsy	*N*	Blood GWA	*N*	%	Events	Hazard ratio	95% CI	*P*
ARSI	Failure free survival (FFS)	At baseline (BL)	154	GWAlow	78	51%	61	1.00		
				GWAhigh	76	49%	75	2.63	1.86–3.72	< 0.001
		At early timepoint (ET)	40	GWAlow	31	78%	27	1.00		
				GWAhigh	9	22%	9	6.73	2.60–17.42	< 0.001
		Longitudinal (BL & ET)^a^	40	GWAlow	20	50%	16	1.00		
				GWAswitch	11	28%	11	1.36	0.63–2.96	0.43
				GWAhigh	9	22%	9	7.61	2.77–20.88	< 0.001
Taxanes	Time to next line of treatment (TTNT)	At baseline (BL)	42	GWAlow	23	55%	23	1.00		
				GWAhigh	19	45%	19	1.01	0.54–1.88	0.98
		At early timepoint (ET)	42	GWAlow	30	71%	30	1.00		
				GWAhigh	12	29%	12	2.79	1.34–5.78	0.006
		Longitudinal (BL & ET)^a^	42	GWAlow	21	50%	21	1.00		
				GWAswitch	11	26%	11	0.72	0.33–1.54	0.39
				GWAhigh	10	24%	10	2.35	1.07–5.15	0.032

^a^ Dynamics is combined analyses of GWA status at baseline (BL) and at early timepoint (ET) together: GWA Low (GWA < 5) or GWA High (GWA ≥ 5) at both timepoints, or GWAswitch with GWA Low at BL and High at ET (*n* = 0 for ARSI, *n* = 2 for Taxanes) or GWA High at BL and Low at ET (*n* = 11 for ARSI, *n* = 9 for Taxanes).

**Table 3:** Interaction analysis of outcome.

	Hazard ratio	95% CI	*P*
At baseline			
Treatment type (ARSI, Taxanes)	2.36	1.43–3.88	0.001
GWA status (High, Low)	1.08	0.59–1.98	0.81
Interaction (GWA status × treatment type)	2.58	1.28–5.20	0.008
At early timepoint			
Treatment type (ARSI, Taxanes)	1.77	1.03–3.04	0.039
GWA status (High, Low)	2.43	1.21–4.86	0.012
Interaction (GWA status × treatment type)	3.53	1.24–10.01	0.018

To analyze the dynamics of GWA status, we combined the baseline and early time point samples. This showed that patients with GWA^low^ and patients with disconcordant GWA at both time points, i.e. GWA switching [from GWA^high^ to GWA^low^ (*n* = 9), or from GWA^low^ to GWA ^high^ (*n* = 2), vs. patients with GWA^high^ at both time points, showed significant differences in TTNT for taxanes (*P* < 0.04) and in FFS for ARSI treatment (*P* < 0.001) (Fig. 4 and Table 2)].

## 4 Discussion

Whilst treatment options in mCRPC patients have expanded, it remains hard to adequately determine treatment response. Traditional markers and current clinical tools for mCRPC have limitation in assessing response and do not accurately predict disease progression before radiographic assessments at 3 and/or 6 months [7]. Detection of ctDNA has arrived as a predictive or prognostic biomarker capable of noninvasive treatment monitoring [8, 9, 10, 11, 12, 13]. Recently, we reported that the mFast‐SeqS method was able to quantify ctDNA levels yielding prognostic value at baseline in mCRPC patients [17]. Whether a mFast‐SeqS‐derived GWA score, or dynamic changes of this measure can also serve as a treatment response marker was still up for debate. In the current study, we investigated whether a dichotomized GWA score derived by the mFast‐SeqS method in cfDNA at the start of a new treatment and/or early time point could serve as a treatment response marker and whether the GWA score correlated with WGS from metastatic tumor biopsies. Our findings showed that at an early time point, dichotomized GWA scores were related with response in patients treated with ARSI and with taxanes. The GWA score at baseline was significantly associated with ARSI treatment length in patients treated with ARSI; however, GWA scores were unable to determine TTNT in patients treated with taxanes. This difference was significant in an interaction test suggesting baseline GWA score has predictive value.

Another observation we made was that by combining baseline data with data from the early time point in ARSI treated patients, it appeared discriminative power of GWA scoring increased as the hazard ratio increased from 2.63 at baseline to 7.61 in the combined measure. Although our observations will require further validation in independent cohorts, we can conclude that this minimally invasive, user‐friendly, affordable and robust mFast‐SeqS assay measure might have valuable clinical value. On top of that, it was reassuring that our reported GWA scores in cfDNA correlated well with corresponding tumor‐tissue chromosomal profiles. Interestingly, patients with GWA^high^ score compared to GWA^low^ had a remarkably higher number of structural variants in their tumor tissue. Thus, we find a group of patients with a GWA^high^ score pinpointing patients having increased genomic instability.

A limitation of this study is the lack of additional clinical data such as PSA values, known prognostic markers such as ECOG, metastatic burden, neuroendocrine differentiation, International Society of Urological Pathology (ISUP) grade or imaging data. To determine whether GWA scores at early on treatment time point would be a treatment response marker, comparison with PSA values or imaging data as reference is recommended. Another potential weakness of this study was that GWA score threshold, i.e. < 5 and ≥ 5 used for the dichotomization of aneuploidy status was based on previous literature [14]. The threshold for grouping patients might be improved to better optimize the stratification; however, this remains to be shown. Finally, the mode to evaluate GWA dynamics was also exploratory and the study design (not a randomized controlled trial), limited us from truly assessing its predictive value. The low numbers of patients treated with cabazitaxel in this study could have limited us from finding prognostic value at baseline in these patients. Although our study was not randomized, the taxane cohort was small and we previously reported prognostic value of cabazitaxel treated patients [22], we now report that the aneuploidy score is an early response marker.

## 5 Conclusion

In conclusion, mFast‐SeqS‐based GWA scores are concordant with aneuploidy scores obtained by WGS from tumor‐tissue and can predict response to ARSI treatment at baseline and, at early time point, to ARSI and taxanes. This assay can be easily performed at low cost, requiring little input of cfDNA. Ultimately, if further validated, these results can improve the cost‐effectiveness of a treatment by sparing patients ineffective treatments and, at the same time, unnecessary toxicity.

## Conflict of interest

JWMM declares a consultancy fee by Novartis, and institutional research grants by Pfizer and GSK (all outside the submitted work). PH has received consulting or advisory fees from Astellas, MSD, Pfizer AstraZeneca, BMS and Ipsen. MPL received institutional grants from JnJ, Astellas, MSD and Sanofi, consulting fees from Sanofi, Johnson & Johnson, Merck, Astellas, Incyte, Amgen, Janssen Cilag, Bayer, Servier and Pfizer, and is currently employed by Amgen. RW has advisory or speakers fees for Astellas, Merck, Bayer and received institutional research grants from Bayer. MSH has received institutional research funding from Bristol Myers Squibb, 4SC, Roche, Astellas Pharma Netherlands and AstraZeneca, and institutional consultancy fees from Bristol‐Myers Squibb, Roche, Merck Sharp & Dohme, AstraZeneca, Pfizer, Janssen, and Seattle Genetics. The remaining authors declare no conflict of interest.

## Author contributions

Study concept and design: MPL, SMW, RW, MPHMJ, JWMM. Acquisition of data: All authors. Analysis and interpretation of data: KTI, ACJ, JANG, JR, SMW, MPHMJ, JWMM, NB. Drafting of the manuscript: KTI, MPHMJ, JWMM. Critical revision of the manuscript for important intellectual content: All authors. Statistical analysis: KTI, JR, MPHMJ. Obtaining funding: MPL, RW, MPHMJ, JWMM. Administrative, technical, or material support: KTI, ACJ, LFD, VW, CB, JH, DV, SMW, MPHMJ, PH, MSH. Supervision: MPHMJ, JWMM. Other: None.

## Peer review

The peer review history for this article is available at https://www.webofscience.com/api/gateway/wos/peer‐review/10.1002/1878‐0261.13797.

## Supporting information


**Fig. S1.** Correlation plot between *Z*‐score and CN.

**Fig. S2.** Correlation plot between GWA score and CNA in tumor tissue.

**Fig. S3.** Digital PCR AR/AGO1 CN‐ratio.

**Fig. S4.** Digital PCR AR/AGO1 versus FastSeq GWA.

## Data Availability

The WGS and corresponding clinical data used in this study were made available by the Hartwig Medical Foundation (HMF; Dutch nonprofit biobank organization) and provided under data request number DR‐170. Therefore, the data are available under restricted access and can be requested by contacting the HMF (https://www.hartwigmedicalfoundation.nl). The remaining data are available upon request to the corresponding author.
