# Supplementary figures and images for: Cell‐free DNA aneuploidy score as a dynamic early response marker in prostate cancer
Source: Mol Oncol. 2025 Mar 14;19(10):2822–32. doi: 10.1002/1878-0261.13797 (PMC12515704; doi:10.1002/1878-0261.13797)

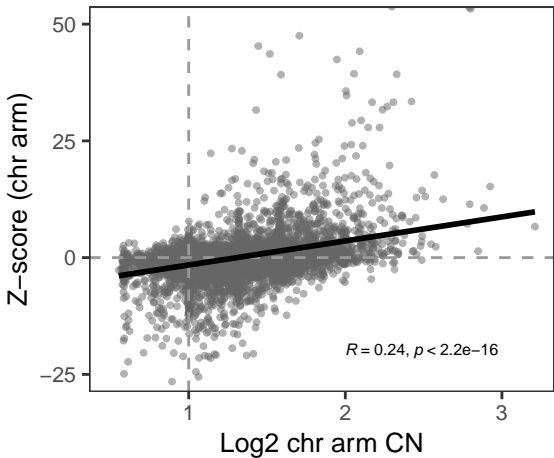

Supplement: Supplementary file 1 — Fig. S1. Correlation plot between Z‐score and CN. [file MOL2-19-2822-s004.pdf]

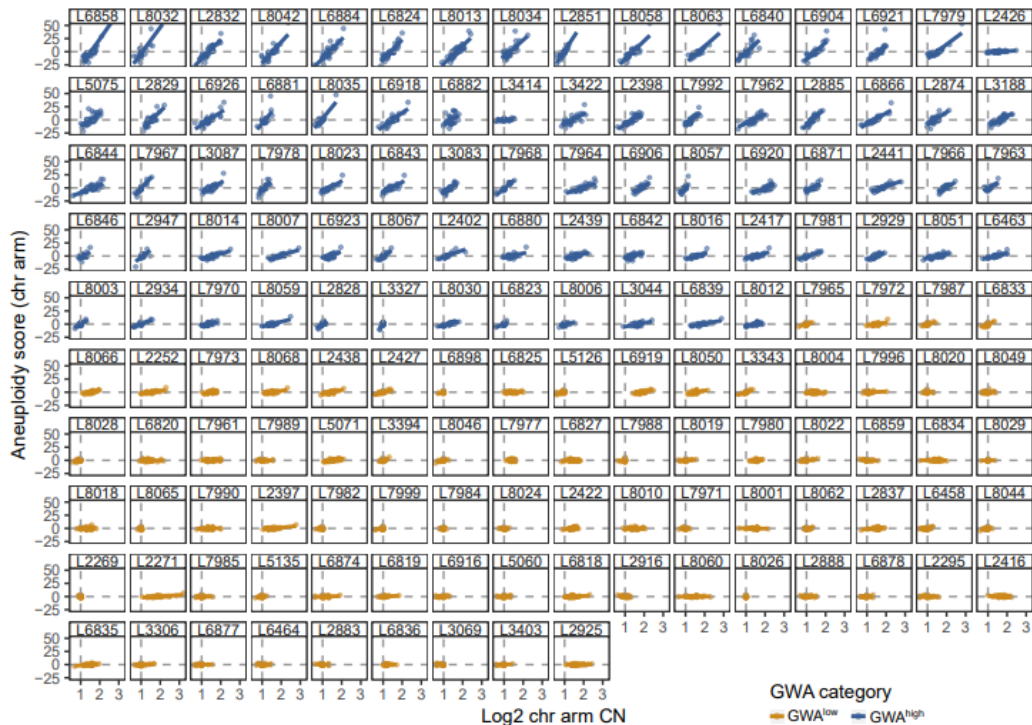

Supplement: Supplementary file 2 — Fig. S2. Correlation plot between GWA score and CNA in tumor tissue. [file MOL2-19-2822-s001.pdf]

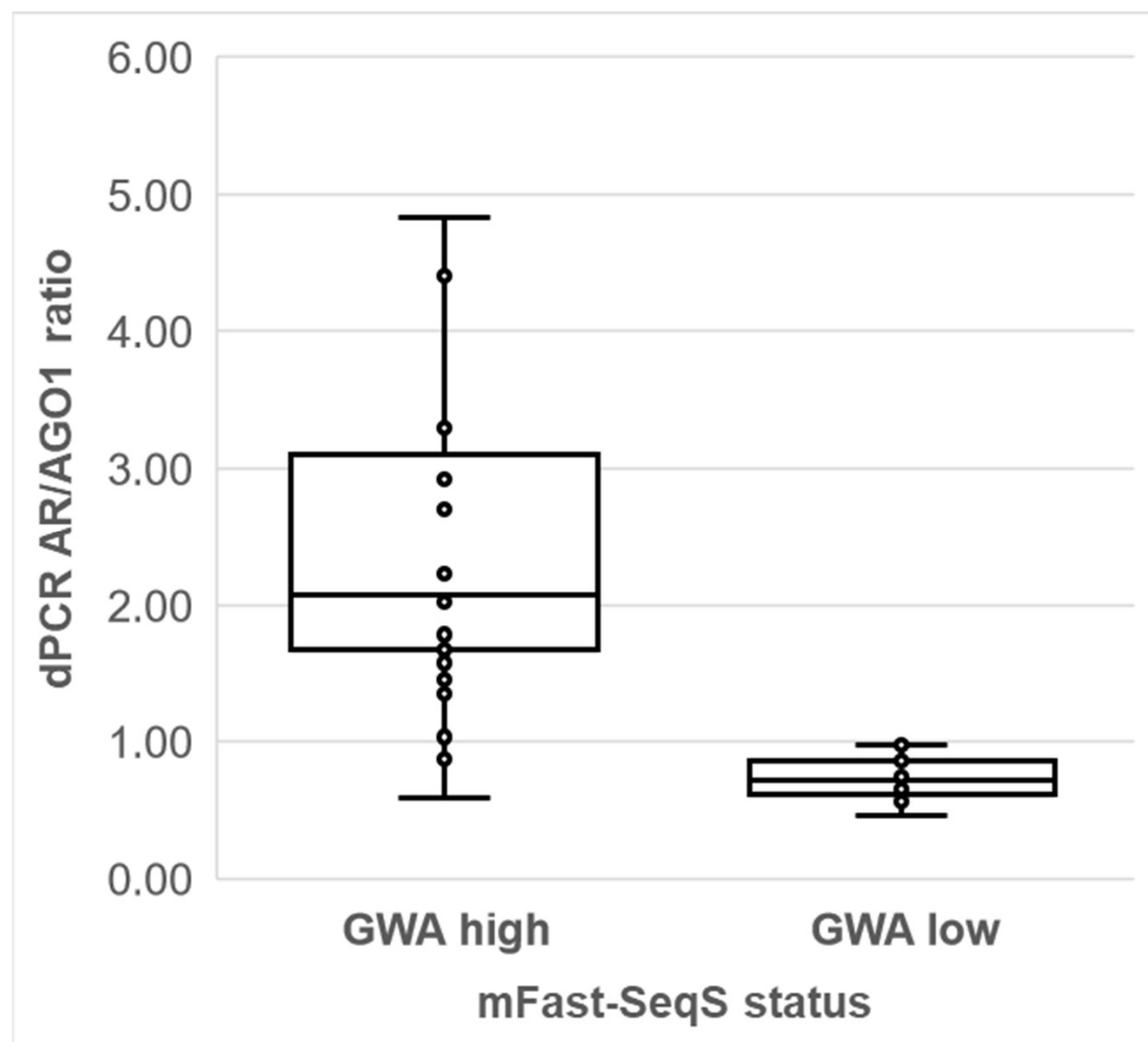

Supplement: Supplementary file 3 — Fig. S3. Digital PCR AR/AGO1 CN‐ratio. [file MOL2-19-2822-s003.pdf]

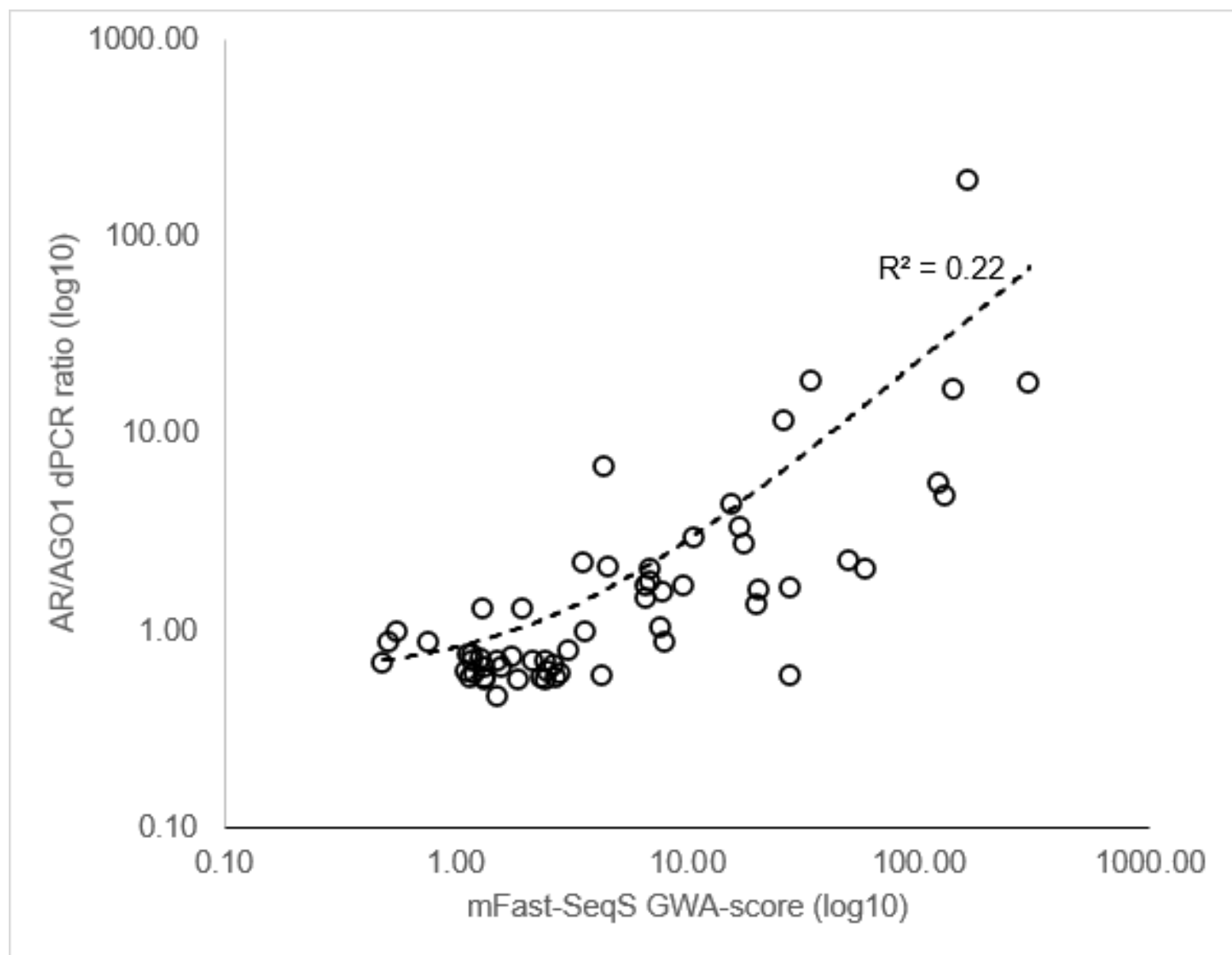

Supplement: Supplementary file 4 — Fig. S4. Digital PCR AR/AGO1 versus FastSeq GWA. [file MOL2-19-2822-s002.pdf]
